# Childhood adversity and educational attainment: Evidence from Zambia on the role of personality

**DOI:** 10.3389/fpsyg.2023.995343

**Published:** 2023-01-27

**Authors:** Peter C. Rockers, Stephanie Simmons Zuilkowski, Günther Fink

**Affiliations:** ^1^Department of Global Health, Boston University School of Public Health, Boston, MA, United States; ^2^Department of Educational Leadership and Policy Studies, Florida State University, Tallahassee, FL, United States; ^3^Swiss Tropical and Public Health Institute, Basel, Switzerland; ^4^University of Basel, Basel, Switzerland

**Keywords:** childhood adversity, personality traits, educational attainment, Zambia, death of a parent

## Abstract

**Introduction:**

We examine whether personality traits mediate the association between childhood adversity and educational attainment using longitudinal data from a cohort in Zambia.

**Methods:**

We fit a structural equation model using data on three forms of childhood adversity—household poverty, stunting as a measure of chronic malnutrition, and death of a parent—and data on the “Big Five” personality traits and educational attainment assessed at 15 years of age.

**Results:**

We find that childhood poverty and death of a parent are associated with lower openness to experience. Furthermore, openness to experience mediates 93% of the negative association between death of a parent and school enrollment and 19% of the negative association between childhood poverty and enrollment.

**Discussion:**

Our findings reinforce a diverse and growing body of evidence linking childhood adversity to educational attainment while also placing it in a new light. Future work should continue to examine the biological and psychosocial pathways that determine openness to experience and other personality traits, as well as their role in shaping important life outcomes.

## 1. Introduction

A large and growing body of evidence has linked childhood adversity to lower educational attainment (Montez and Hayward, 2014). Children who grow up in poverty drop out of school earlier on average in low- and middle-income countries (LMICs; Filmer and Pritchett, 1999) and are less likely to attend post-secondary school in high-income countries (HICs; Haveman et al., 1991). Having limited household resources results in financial barriers to enrollment, while growing up in a culture of poverty appears to affect attitudes toward schooling, which may contribute to earlier dropout (Vaisey, 2010). Poor health in childhood is also associated with lower educational attainment, though more work is needed to clarify causal pathways that may determine this relationship (Palloni, 2006; Jackson, 2009). Finally, death of a parent has been shown to negatively affect educational attainment (Ainsworth and Filmer, 2006; Case and Ardington, 2006; Evans and Miguel, 2007), likely due to the long-term psychological effects of bereavement (Tremblay and Israel, 1998) as well as lost household wealth (Case et al., 2004).

Evidence spanning several disciplines suggests that personality traits may mediate the relationship between childhood adversity and educational attainment. An emerging psychology literature finds that childhood adversity exerts a strong influence on the development of personality traits (Carver et al., 2014; Fletcher and Schurer, 2017; Davis et al., 2018; Conger et al., 2021). The influence of adversity likely manifests through biological and psychosocial pathways, with genetics playing an important but not wholly determining role (McCrae et al., 2000). Adversity experienced in childhood can become biologically embedded and change the structure and function of the brain (Nelson et al., 2020), and evidence from personality neuroscience suggests that these changes can influence emerging personality traits (DeYoung et al., 2010). Adversity also affects the family and social environment in which children grow up, which likely affects personality through social development processes (Caspi et al., 2005).

A robust literature spanning education research and empirical economics links personality to schooling outcomes (De Raad and Schouwenburg, 1996). Studies have demonstrated a consistent and strong relationship between personality and educational attainment, including graduation from high school (Heckman et al., 2006; Nandi and Nicoletti, 2014). A recent meta-analysis found that key personality traits are strongly correlated with academic performance (Poropat, 2009). Most of the research on this topic has been conducted in HICs and it is not clear whether the relationship between personality and schooling holds consistently in LMICs. One recent study that used data from a cohort in rural China found that aspects of early adolescent personality predicted eventual educational attainment (Glewwe et al., 2017).

A recent study of the long-term effects of the Perry Preschool Program in the United States is one of the first to bridge the literatures on early childhood adversity, personality, and educational outcomes (Heckman et al., 2013). Using an experiment initiated in Michigan in the 1960s, the study found strong evidence that the program, an innovative preschool curriculum delivered from 3 to 5 years of age (Schweinhart and Weikart, 1981), affected personality and that personality mediated the effects of the program on educational performance in adolescence and on other adult outcomes, including income.

### 1.1. Childhood adversity

This study focuses on three forms of childhood adversity that have been shown to be associated with educational attainment: household poverty (Haveman et al., 1991; Filmer and Pritchett, 1999; Montez and Hayward, 2014), chronic malnutrition (Palloni, 2006; Jackson, 2009), and death of a parent (Ainsworth and Filmer, 2006; Case and Ardington, 2006; Evans and Miguel, 2007). Early exposure to these forms of adversity are a subset of potential threats to child well-being (Ben-Arieh et al., 2014). In 2010, when data on childhood adversity were collected for this study, 61% of Zambian households were below the international poverty line (World Bank, 2020). In this analysis, we construct a measure of household poverty based on a household asset index. Previous research in Zambia using a similar asset measure has shown that children raised in lower-wealth households are more likely to experience food insecurity (Bulawayo et al., 2019) and to suffer from malnutrition (Mzumara et al., 2018). They also have reduced access to clean water and sanitation [UNICEF (United Nations Children’s Fund) and WHO (World Health Organization), 2019] and experience more frequent illness, including diarrhea (Zambia Statistics Agency et al., 2019) and malaria (Zambia Ministry of Health, 2018). We use stunting status as a measure of chronic malnutrition. Stunting in childhood reflects the cumulative effects of several facets of nutrition and health and is influenced by exposures *in utero*, nutrition during childhood, and acute and chronic illnesses (Perumal et al., 2018). In Zambia, stunting and childhood health more generally reflect household wealth but may exert an independent influence on educational attainment (McCoy et al., 2015). Finally, we examined the effects of death of a parent during childhood, which participants in this study experienced at a relatively high rate due to the HIV epidemic. Recruitment for the study occurred at households and as a result, nearly all study children who experienced the death of a parent resided with family rather than in an orphanage.

### 1.2. Personality

Personality traits are “individual differences in characteristic patterns of thinking, feeling and behaving” [APA (American Psychological Association), 2021]. Formal scientific study of personality has been ongoing for a century, at least (Allport, 1937). Over the past few decades, there has been increasing convergence around a set of conceptual frameworks and psychometrics for assessing individual personality (McCrae and Costa, 1997). The most common taxonomy of personality traits used in empirical social science research is the “Big Five,” which defines five traits: openness to experience (openness hereafter), conscientiousness, extraversion, agreeableness, and neuroticism (Digman, 1990). While research on personality traits in sub-Saharan Africa is limited, there is consistent evidence that the Big Five model is valid among many African populations (Zecca et al., 2013; Thalmayer and Saucier, 2014), though alternative models may be more appropriate for highly structured, traditional societies (Thalmayer et al., 2020).

Personality traits emerge from a set of complex interactions between genetic and environmental factors (Bouchard, 1994). A large body of empirical evidence suggests that the roots of personality are expressed as temperament during early childhood (Rothbart et al., 2000) and that temperament is strongly influenced by genetics (Zwir et al., 2020). Personality traits continue to change over the life course, becoming more stable at older ages (Roberts and DelVecchio, 2000; Caspi et al., 2005). Personality traits predict a diverse set of outcomes across a variety of domains including education (De Raad and Schouwenburg, 1996), health (Booth-Kewley and Vickers, 1994; Reiss et al., 2014), employment (Nyhus and Pons, 2005), and politics (Caprara et al., 2006). Research on human capital formation increasingly identifies personality traits as capabilities that are as important to life outcomes as traditional skills like literacy and numeracy (Cunha and Heckman, 2007; Borghans et al., 2008).

### 1.3. Educational attainment

Higher educational attainment is associated with better labor market outcomes, including higher income (Patrinos and Psacharopoulos, 2020). For many Zambians, completing secondary school may create a path out of poverty that would otherwise be unavailable. There is also a robust literature linking educational attainment with better adult health outcomes, including reduced mortality risk (Pradhan et al., 2017). We examine educational attainment based on data collected when participants were 15 years old. Many participants were still enrolled in school at the time, so we are not able to assess final educational attainment for all participants. We instead focus on enrollment status and “on-track” enrollment status at the time of data collection. According to guidelines published by the Zambia Ministry of Education, 15-year old students should be enrolled in secondary school in order to be on-track to graduate by 18 years of age. We also examine self-reported performance at school as an outcome.

### 1.4. Study aim

We examine the role of personality traits in mediating the association between childhood adversity and educational attainment using longitudinal data from a cohort in Zambia. We hypothesize that there are significant indirect effects of early adversity on educational outcomes through personality pathways. Our findings are relevant to a diverse set of disciplines and fields of study, including psychology, neuroscience, economics, education, and public health. This work also contributes to understanding of personality traits in sub-Saharan Africa, an important but understudied area of inquiry.

## 2. Materials and methods

### 2.1. Sample

Study participants were enrolled in the Zambia Early Childhood Development Project (ZECDP) in 2010. An original sample of 1,686 children born in 2004 was drawn from six of Zambia’s nine provinces (at the time; Zambia now has 10 provinces). Details of the cohort have been published elsewhere (Fink et al., 2012). In 2019, a subset of cohort children residing in three provinces—Lusaka, Eastern, and Luapula—were visited for a follow-up assessment. These provinces were selected because they were part of a previous sub-study that was nested within the ZECDP and additional data were available for study households (Fink and Rockers, 2017).

### 2.2. Data collection

Data from two time points were analyzed. Household demographics and measures of childhood adversity were collected during the initial interview conducted at the home at the time of enrollment in the ZECDP in 2010. The ZECDP was approved by institutional review boards at the Harvard School of Public Health and the University of Zambia. Personality traits and schooling outcomes were measured during follow-up interviews conducted at the home in 2019. The follow-up study was approved by institutional review boards at the Swiss Tropical and Public Health Institute and the University of Zambia. Participants provided informed consent prior to each survey.

### 2.3. Measures

#### 2.3.1. Childhood adversity

In this analysis, we construct a measure of household poverty based on a household asset index. On average, 5% of observations had missing information for asset variables; for these observations, values were imputed as the sample mean prior to running PCA (Vyas and Kumaranayake, 2006). Household poverty was defined as being in the bottom 61% of the full ZECDP sample based on the wealth index, consistent with the World Bank’s estimate of the poverty rate in Zambia at the time of data collection (World Bank, 2020). Height data were converted to height-for-age *z*-scores (HAZ) using the World Health Organization growth reference data for 5–19 years [WHO (World Health Organization), 2021]. Children with HAZ < −2 were defined as stunted. Death of a parent was reported by the child’s primary caregiver at the time of the initial household survey and was coded as a dichotomous variable that did not distinguish whether the child had lost one or both parents.

#### 2.3.2. Personality traits

Personality traits were measured using the short form of the Big Five Inventory (BFI-S; John and Srivastava, 1999), a 15-item instrument based on the original 44-item BFI (Gerlitz and Schupp, 2005), that was developed for use in the German Socio-Economic Panel Study. The BFI-S has demonstrated high validity among populations in high-income countries (Lang et al., 2011). One validation study conducted in Germany (Hahn et al., 2012) reported the following reliability coefficients (Cronbach’s alpha): neuroticism = 0.66; extraversion = 0.76; openness = 0.58; agreeableness = 0.44; and conscientiousness = 0.60. That same study found that the five-factor model explained 62% of the total variance in the sample, with a high mean first loading (0.74) and a low mean second loading (0.12). The World Bank has validated the BFI-S within its STEP skill measurement surveys, which have been administered in LMICs throughout the world (Pierre et al., 2014). A recent validation study of the STEP data (Laajaj et al., 2019) reported the following reliability coefficients (Cronbach’s alpha): neuroticism = 0.53; extraversion = 0.51; openness = 0.50; agreeableness = 0.46; and conscientiousness = 0.47. We administered the version of the BFI-S used in the STEP surveys without further adaptation.

The BFI-S includes three items for each of the five personality traits (see Supplemental materials). Response options (and scoring) for each item were: “almost always” (4); “most of the time” (3); “some of the time” (2); and “almost never” (1). Raw trait scores were generated by summing responses for the three items that corresponded to that trait. For three of the 15 items, a response of “almost always” indicates a lower level of the trait; responses to these items were reverse coded prior to generating raw trait score. For analysis, raw trait scores were converted to z-scores by standardizing within the study population.

#### 2.3.3. Educational attainment

Respondents reported whether they were enrolled in school at the time of the follow-up assessment. On-track enrollment was defined as enrollment in secondary school. In addition, all respondents, regardless of school enrollment status, self-reported their overall academic performance as “below average,” “average,” or “above average.” Responses were dichotomized for analysis as “above average” versus “average” or “below average.”

### 2.4. Analysis

**Figure 1:**
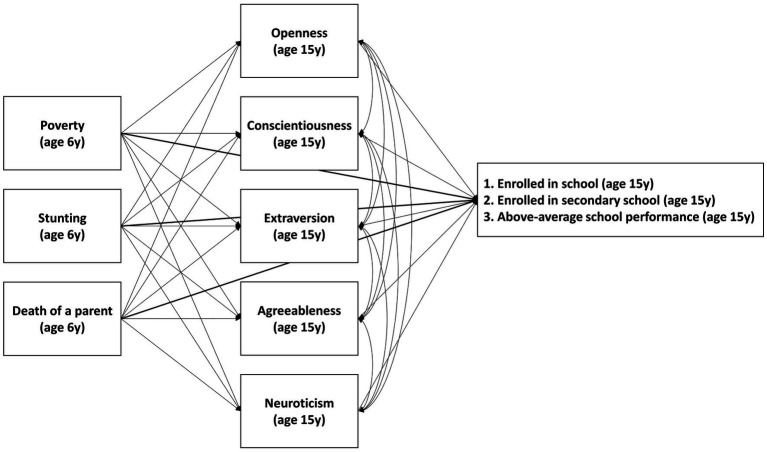
Diagram of structural equation models. *Notes:* Straight lines indicate modeled pathways and curved lines indicate modeled residual covariance.

First, we summarized demographic variables including the child’s gender and age in months at both rounds of data collection, along with the childhood adversity and schooling measures described above. Second, we conducted a set of path analyses by fitting the structural equation model described in Figure 1 for each of the three educational outcomes to estimate: (1) associations between childhood adversity and personality traits; (2) associations between personality traits and educational outcomes; (3) direct associations between childhood adversity and educational outcomes; and (4) indirect associations between childhood adversity and educational outcomes mediated *via* personality traits. Only data from participants enrolled in school were included in the model with on-track enrollment as the outcome. All models consisted entirely of observed variables and were specified as saturated, i.e., with zero degrees of freedom, and as such did not produce meaningful goodness-of-fit statistics (Agler and De Boeck, 2017). All models were fit using the *lavaan* package in R. Standard errors were adjusted using the Huber-White sandwich estimator to account for clustered sampling in the original study design.

## 3. Results

We analyzed data from 315 participants assessed at two waves of data collection (Table 1). Participants were 74.3 months (6.2 years) old on average at the initial assessment and 184.0 months (15.3 years) on average at the follow-up assessment. At the time of the initial assessment, 20.0% resided in a household with electricity and 9.8% in households with running water. Overall, 78.1% of children resided in poverty, 19.4% were stunted, and 6.4% experienced the death of a parent. At the time of the follow-up, 79.0% were still enrolled in school, but only 42.2% were enrolled in secondary school. Around one-third of participants rated their academic performance as above average. Compared to the full ZECDP sample, the study sample had higher rates of childhood poverty and lower rates of parent death (see Supplemental materials).

**Table 1:** Description of study participants (*N* = 315).

	*n* (%)
*Demographics*	
Female	163 (51.8)
Age at initial assessment (months), *mean* (*SD*)	74.3 (4.5)
Age at follow-up assessment (months), *mean* (*SD*)	184.0 (3.6)
Province	
Lusaka	100 (31.8)
Luapula	101 (32.1)
Eastern	114 (36.2)
*Childhood adversity*	
Key household assets	
Electricity	63 (20.0)
Running water	31 (9.8)
Bed	188 (59.7)
Poverty	246 (78.1)
Stunting	61 (19.4)
Death of a parent	20 (6.4)
*Schooling*	
Enrollment in school at follow-up	
Not enrolled	66 (21.0)
Enrolled in primary school	116 (36.8)
Enrolled in secondary school	133 (42.2)
Above average academic performance (self-report)^a^	98 (31.1)

a Data on academic performance were missing for 20 respondents.

Poverty (β −0.502; *p* < 0.001) and death of a parent (β −0.494; *p* < 0.001) were negatively associated with openness (Table 2), while stunting was negatively associated with neuroticism (β −0.210; *p* = 0.025).

**Table 2:** Effect of childhood adversity on personality.

	Personality				
	Open.	Cons.	Extra.	Agree.	Neuro.
Poverty	−0.502*** (0.119)	0.068 (0.151)	0.056 (0.134)	−0.019 (0.173)	−0.014 (0.141)
Stunting	−0.087 (0.139)	0.093 (0.108)	0.060 (0.179)	0.030 (0.172)	−0.210* (0.093)
Death of a parent	−0.494*** (0.141)	−0.060 (0.248)	−0.064 (0.263)	−0.105 (0.202)	0.249 (0.187)

Numbers in parentheses are adjusted standard errors.

* *p* < 0.05.

*** *p* < 0.001.

Openness was positively associated with enrollment in school (β 0.076; *p* = 0.008; Table 3), while agreeableness was negatively associated (β −0.055; *p* = 0.012). Poverty had a negative direct association with enrollment (β −0.162; *p* = 0.029) and a negative indirect association mediated *via* openness (β −0.038; *p* = 0.027). Death of a parent was not directly associated with enrollment but had a negative indirect association mediated *via* openness (β −0.037; *p* = 0.033). Stunting was not directly or indirectly associated with enrollment. Openness mediated 93% of the total association between death of a parent and enrollment in school and 19% of the total association between poverty and enrollment.

**Table 3:** Direct and indirect effects on educational outcomes.

	Total effect	Direct effect	Indirect effect *via* personality				
			Open.	Cons.	Extra.	Agree.	Neuro.
**Panel A. Outcome: enrollment in school (*n* = 315)**							
Childhood adversity							
Poverty	−0.198** (0.063)	−0.162* (0.074)	−0.038* (0.017)	0.001 (0.002)	0.000 (0.001)	0.001 (0.010)	0.000 (0.001)
Stunting	−0.023 (0.079)	−0.014 (0.083)	−0.007 (0.012)	0.001 (0.002)	0.000 (0.002)	−0.001 (0.009)	−0.001 (0.004)
Death of a parent	−0.040 (0.097)	−0.009 (0.101)	−0.037* (0.018)	−0.001 (0.002)	0.000 (0.002)	0.006 (0.011)	0.001 (0.005)
Personality							
Openness (*z*-score)	0.076** (0.028)						
Conscientiousness (*z*-score)	0.010 (0.020)						
Extraversion (*z*-score)	−0.004 (0.027)						
Agreeableness (*z*-score)	−0.055* (0.022)						
Neuroticism (*z*-score)	0.005 (0.019)						
**Panel B. Outcome: On-track enrollment in school (*n* = 249; sample excludes those not enrolled in school)**							
Childhood adversity
Poverty	−0.306*** (0.088)	−0.302*** (0.091)	−0.013 (0.013)	0.002 (0.006)	0.003 (0.005)	0.004 (0.008)	0.001 (0.008)
Stunting	−0.230*** (0.059)	−0.242*** (0.062)	−0.003 (0.005)	0.003 (0.005)	0.000 (0.007)	−0.001 (0.007)	0.013 (0.009)
Death of a parent	−0.053 (0.078)	−0.020 (0.086)	−0.017 (0.015)	−0.006 (0.012)	−0.004 (0.012)	0.009 (0.012)	−0.015 (0.013)
Personality							
Openness (*z*-score)	0.034 (0.028)						
Conscientiousness (*z*-score)	0.035 (0.027)						
Extraversion (*z*-score)	0.038 (0.024)						
Agreeableness (*z*-score)	−0.041 (0.029)						
Neuroticism (*z*-score)	−0.047^†^ (0.028)						
**Panel C. Outcome: above average school performance (self-report; *n* = 295)**							
Childhood adversity							
Poverty	0.060 (0.123)	0.111 (0.125)	−0.053 (0.022)*	0.004 (0.008)	−0.002 (0.004)	0.000 (0.000)	0.000 (0.001)
Stunting	0.004 (0.050)	0.016 (0.055)	−0.015 (0.020)	0.003 (0.006)	−0.002 (0.004)	0.000 (0.002)	−0.002 (0.007)
Death of a parent	−0.152^†^ (0.092)	−0.109 (0.091)	−0.050 (0.023)*	−0.003 (0.014)	0.000 (0.006)	0.000 (0.006)	0.004 (0.014)
Personality							
Openness (*z*-score)	0.117*** (0.037)						
Conscientiousness (*z*-score)	0.052 (0.036)						
Extraversion (*z*-score)	−0.025 (0.021)						
Agreeableness (*z*-score)	0.000 (0.031)						
Neuroticism (*z*-score)	0.009 (0.030)						

Numbers in parentheses are adjusted standard errors. On-track enrollment was defined as enrollment in secondary school at the time of the follow-up (age 15).

† *p* < 0.1.

* *p* < 0.05.

** *p* < 0.01.

*** *p* < 0.001.

Both poverty (β −0.302; *p* = 0.001) and stunting (β −0.242; *p* < 0.001) were negatively associated with on-track enrollment among respondents enrolled in school. Personality traits were not associated with on-track enrollment and did not mediate any indirect associations between adversity and on-track enrollment.

Finally, openness was positively associated with academic performance (β 0.117; *p* = 0.001). While none of the measures of childhood adversity were directly associated with performance, poverty (β −0.053; *p* = 0.015) and death of a parent (β −0.050; *p* = 0.032) were indirectly associated with performance *via* openness.

## 4. Discussion

We examined the role of personality in mediating the association between childhood adversity and educational attainment using longitudinal data from a cohort in Zambia. Our analysis yielded three main findings. First, poverty and death of a parent were negatively associated with openness. Second, consistent with previous research, openness was positively associated with school enrollment and academic performance (De Raad and Schouwenburg, 1996). Openness is thought to reflect intellectual curiosity and mental fluidity (Moutafi et al., 2006), and individuals with a higher degree of openness likely derive greater satisfaction from being in a school environment and from being challenged by school curricula. Third, openness mediated nearly the entire negative association between death of a parent and enrollment as well as 19% of the total association between poverty and enrollment. Openness also mediated the negative associations of poverty and death of a parent with academic performance. This finding confirms our main hypothesis that there are significant and rather large indirect effects of early adversity on educational outcomes, acting through personality pathways.

Our findings reinforce a diverse and growing body of evidence linking childhood adversity to educational attainment while also placing it in a new light (Haveman et al., 1991; Montez and Hayward, 2014). It appears that adverse experiences may alter personality trajectories, contributing to negative outcomes later in life. The predominant role of openness in mediating the relationship between death of a parent and school enrollment is particularly striking and is consistent with previous research conducted in South Africa that concluded that “scarring” likely plays an important role in determining the relationship (Case and Ardington, 2006). As demonstrated by the Perry Preschool Program (Heckman et al., 2013), early-life interventions that mitigate adversity could generate significant benefits in part *via* personality pathways (Almlund et al., 2011; Black et al., 2017). In LMICs, anti-poverty programs that target resources to young children may be effective in this regard. Additional support services may be required for children who have experienced the death of a parent early in life, an issue of particular importance in countries with large orphan populations as a result of the HIV epidemic.

Whether childhood adversity influences personality primarily through biological or psychosocial mechanisms remains an open question. Personality psychologists have observed that the characteristics that define openness are closely linked to cognitive processes (DeYoung, 2015); the trait is sometimes referred to as “intellect” or “openness/intellect.” Empirical neuroscience has demonstrated a strong association between openness and brain structure and function (Dubois et al., 2018). The negative effects of poverty on child neurodevelopment are well-documented (Blair and Raver, 2012) and biological mechanisms may be particularly important in explaining the mediating role of personality in the poverty pathway.

Death of a parent may similarly influence personality through biological mechanisms, particularly through the activation of stress response systems (Luecken and Roubinov, 2012), but psychosocial mechanisms are likely also important. Studies have found that children who lose a parent are at higher risk of developing depression and other psychopathology and functional impairment (Brent et al., 2009; Melhem et al., 2011). Similar forms of acute trauma during childhood have been found to be important in the development of personality disorders (Zanarini and Frankenburg, 1997), though more work is needed to understand their role in personality formation generally. Most studies on childhood bereavement and its effects have been conducted in HICs and there is limited evidence from LMICs like Zambia.

Our findings could have implications for practitioners working with children in LMICs like Zambia, including psychologists, health professionals, and teachers. Personality is determined in part by experience and appears to be malleable to change with new experiences, including targeted interventions (Stieger et al., 2021). There may be opportunities to provide support services to children who experience early adversity that, for example, increase openness, which could lead to better educational and overall life outcomes.

## 5. Limitations

This analysis had important limitations. First, more work is needed to validate the BFI-S as a measure of personality in LMICs. We conducted a factor analysis and estimated Cronbach’s alphas for each personality trait (see Supplemental materials) and found relatively low internal consistency. Similar results were found in a recent study of the performance of the BFI-S in several LMICs (Laajaj et al., 2019). Second, our measure of academic performance is self-reported and may generate bias if personality influences how individuals interpret objective indicators of their own performance. Third, we measured personality traits and educational outcomes at the same time and therefore cannot empirically establish the temporal precedence of personality. Personality psychologists have demonstrated that the roots of personality begin to form as early as age 3 and are moderately stable by age 15 but can continue to change throughout the life course (Caspi et al., 2005). Fourth, our sample size was too small to explore differences in the effects of death of a mother, death of a father, and death of both parents. Previous evidence from South Africa suggests that death of a mother has a stronger negative effect on schooling outcomes (Case and Ardington, 2006). Fifth, we did not collect data from parents on personality traits, which would have allowed for a more robust examination of intergenerational effects. Previous studies have found evidence of intergenerational transmission of personality (Sutin et al., 2017). Intergenerational effects might also manifest as genetic endowments correlated with poverty that generate low returns to schooling (Behrman and Taubman, 1989). In our data, parent education was strongly associated with household poverty and enrollment in school but was not associated with personality after accounting for poverty (data not shown). These findings suggest that intergenerational effects acting outside of the poverty channel may be minimal. Future work on this topic employing experimental methods could serve to strengthen understanding of the relevant causal pathways.

## 6. Conclusion

Openness appears to play an important role in mediating the relationship between childhood adversity and educational attainment in the Zambian cohort studied. Our results contribute to a growing literature that documents the importance of personality in a diverse set of life outcomes across a variety of domains including education (De Raad and Schouwenburg, 1996), health (Booth-Kewley and Vickers, 1994; Reiss et al., 2014), employment (Nyhus and Pons, 2005), and politics (Caprara et al., 2006). Future work should continue to examine the biological and psychosocial pathways that determine openness to experience and other personality traits, as well as their role in shaping adult outcomes. It may also be fruitful to examine successful early-life interventions like the Perry Preschool Program (Heckman et al., 2013) to investigate the extent to which the personality impacts of childhood adversity can be mitigated through targeted child and family support. This paper provides important new evidence on the measurement of personality traits in sub-Saharan Africa. Future work should aim to improve our understanding of the underlying structure of personality in this region of the world and to develop new psychometric tools to measure personality, as appropriate.

## Data availability statement

The datasets presented in this study can be found in online repositories. The names of the repository/repositories and accession number(s) can be found below: Harvard Dataverse (Rockers et al., 2023). Available at doi: 10.7910/DVN/JM3AUH.

## Ethics statement

The studies involving human participants were reviewed and approved by Harvard University IRB, Swiss Tropical and Public Health Institute IRB, University of Zambia Research Ethics Committee. Written informed consent to participate in this study was provided by the participants’ legal guardian/next of kin.

## Author contributions

PR conceptualized the study and methodology, conducted the formal analysis, and wrote the initial draft of the manuscript. SS contributed to the conceptualization and methodology and made significant contributions to the final manuscript. GF led funding acquisition, oversaw project administration, contributed to the conceptualization and methodology, and made significant contributions to the final manuscript. All authors contributed to the article and approved the submitted version.

## Funding

The Zambia Early Childhood Development Project was funded by UNICEF Zambia and the Özyegin Family–AÇEV Global Early Childhood Research Fund through the Center on the Developing Child at Harvard University. The follow-up wave of data collection was funded through the Eckenstein-Geigy Professorship at the Swiss Tropical and Public Health Institute.

## Conflict of interest

The authors declare that the research was conducted in the absence of any commercial or financial relationships that could be construed as a potential conflict of interest.

## Publisher’s note

All claims expressed in this article are solely those of the authors and do not necessarily represent those of their affiliated organizations, or those of the publisher, the editors and the reviewers. Any product that may be evaluated in this article, or claim that may be made by its manufacturer, is not guaranteed or endorsed by the publisher.

## Supplementary material

The Supplementary material for this article can be found online at: https://www.frontiersin.org/articles/10.3389/fpsyg.2023.995343/full#supplementary-material
