# Supplementary material for: Childhood adversity and educational attainment: Evidence from Zambia on the role of personality
Source: Front Psychol. 2023 Jan 27;14:995343. doi: 10.3389/fpsyg.2023.995343 (PMC9912843; doi:10.3389/fpsyg.2023.995343)
Supplement: Supplementary file 1 [file Table_1.pdf]

Table S1 Comparison of study sample and full ZECDP sample

	Study sample (N=315)	Full ZECDP sample (N=1,686)
	**n (%)**	**n (%)**
Demographics		
Female	163 (51.8)	845 (50.1)
Age at initial assessment (months), *mean (SD)*	74.3 (4.5)	74.6 (4.1)
Childhood adversity		
Poverty	246 (78.1)	1,029 (61.0)
Stunting	61 (19.4)	328 (19.5)
Death of a parent	20 (6.4)	196 (11.6)
