# Supplementary material for: Childhood adversity and educational attainment: Evidence from Zambia on the role of personality
Source: Front Psychol. 2023 Jan 27;14:995343. doi: 10.3389/fpsyg.2023.995343 (PMC9912843; doi:10.3389/fpsyg.2023.995343)
Supplement: Supplementary file 2 [file Table_2.pdf]

Table S2 Responses to BFI-S items

Personality trait	Item	Response option	n (%)
Openness	Do you come up with ideas other people haven't thought of before?	Almost always (4)	49 (15.6)
		Most of the time (3)	50 (15.9)
		Some of the time (2)	160 (50.8)
		Almost never (1)	56 (17.8)
	Are you very interested in learning new things?	Almost always (4)	179 (56.8)
		Most of the time (3)	76 (24.1)
		Some of the time (2)	52 (16.5)
		Almost never (1)	8 (2.5)
	Do you enjoy beautiful things, like nature, art and music?	Almost always (4)	144 (45.7)
		Most of the time (3)	83 (26.4)
		Some of the time (2)	70 (22.2)
		Almost never (1)	18 (5.7)
Conscientiousness	When doing a task, are you very careful?	Almost always (4)	133 (42.2)
		Most of the time (3)	111 (35.2)
		Some of the time (2)	64 (20.3)
		Almost never (1)	7 (2.2)
	Do you prefer relaxation more than hard work?	Almost always (1)	40 (12.7)
		Most of the time (2)	43 (13.7)
		Some of the time (3)	122 (38.7)
		Almost never (4)	110 (34.9)
	Do you work very well and quickly?	Almost always (4)	142 (45.1)
		Most of the time (3)	98 (31.1)
		Some of the time (2)	72 (22.9)
		Almost never (1)	3 (1.0)
Extraversion	Are you talkative?	Almost always (4)	16 (5.1)
		Most of the time (3)	25 (7.9)
		Some of the time (2)	140 (44.4)
		Almost never (1)	134 (42.5)
	Do you like to keep your opinions to yourself? Do you prefer to keep quiet when you have a opinion?	Almost always (1)	47 (14.9)
		Most of the time (2)	60 (19.1)
		Some of the time (3)	136 (43.2)
		Almost never (4)	72 (22.9)
	Are you outgoing and sociable, for example, do you make friends very easily?	Almost always (4)	79 (25.1)
		Most of the time (3)	63 (20.0)
		Some of the time (2)	109 (34.6)
		Almost never (1)	64 (20.3)
Agreeableness	Do you forgive other people easily?	Almost always (4)	124 (39.4)
		Most of the time (3)	86 (27.3)
		Some of the time (2)	89 (28.3)
		Almost never (1)	16 (5.1)
	Are you very polite to other people?	Almost always (4)	180 (57.1)
		Most of the time (3)	91 (28.9)
		Some of the time (2)	43 (13.7)
		Almost never (1)	1 (0.3)
	Are you generous to other people with your time or money?	Almost always (4)	106 (33.7)
		Most of the time (3)	83 (26.4)
		Some of the time (2)	110 (34.9)
		Almost never (1)	16 (5.1)
Neuroticism	Are you relaxed during stressful situations?	Almost always (1)	51 (16.2)
		Most of the time (2)	82 (26.0)
		Some of the time (3)	147 (46.7)
		Almost never (4)	35 (11.1)
	Do you tend to worry?	Almost always (4)	62 (19.7)
		Most of the time (3)	45 (14.3)
		Some of the time (2)	147 (46.7)
		Almost never (1)	61 (19.4)
	Do you get nervous easily?	Almost always (4)	55 (17.5)
		Most of the time (3)	45 (14.3)
		Some of the time (2)	142 (45.1)
		Almost never (1)	73 (23.2)
