# Supplementary material for: Childhood adversity and educational attainment: Evidence from Zambia on the role of personality
Source: Front Psychol. 2023 Jan 27;14:995343. doi: 10.3389/fpsyg.2023.995343 (PMC9912843; doi:10.3389/fpsyg.2023.995343)
Supplement: Supplementary file 3 [file Table_3.pdf]

Table S3 Factor loadings for BFI-S items

Personality trait	Item	Factor				
		1	2	3	4	5
**Openness**	Do you come up with ideas other people haven't thought of before?	0.06	0.19	0.26	0.08	0.03
	Are you very interested in learning new things?	-0.06	0.10	0.39	-0.05	0.23
	Do you enjoy beautiful things, like nature, art and music?	-0.03	0.15	0.40	0.15	0.17
**Conscientiousness**	When doing a task, are you very careful?	-0.14	0.14	0.45	-0.18	0.03
	Do you prefer relaxation more than hard work?	0.02	0.11	-0.05	-0.29	-0.11
	Do you work very well and quickly?	-0.12	0.19	0.53	-0.16	-0.09
**Extraversion**	Are you talkative?	0.09	0.03	-0.02	0.19	0.14
	Do you like to keep your opinions to yourself? Do you prefer to keep quiet when you have a opinion?	0.14	-0.05	-0.07	-0.16	0.07
	Are you outgoing and sociable, for example, do you make friends very easily?	-0.01	0.22	0.12	0.13	-0.10
**Agreeableness**	Do you forgive other people easily?	-0.22	0.15	0.37	-0.25	0.09
	Are you very polite to other people?	-0.08	0.14	0.56	-0.11	-0.16
	Are you generous to other people with your time or money?	-0.08	1.00	0.00	0.00	0.00
**Neuroticism**	Are you relaxed during stressful situations?	1.00	0.00	0.00	0.00	0.00
	Do you tend to worry?	-0.14	0.10	0.37	0.45	0.10
	Do you get nervous easily?	-0.04	0.03	0.20	0.29	-0.36
