# Supplementary material for: Childhood adversity and educational attainment: Evidence from Zambia on the role of personality
Source: Front Psychol. 2023 Jan 27;14:995343. doi: 10.3389/fpsyg.2023.995343 (PMC9912843; doi:10.3389/fpsyg.2023.995343)
Supplement: Supplementary file 4 [file Table_4.pdf]

Table S4 Cronbach’s alphas for personality traits

	**Cronbach’s alpha**
**Openness**	0.40
**Conscientiousness**	0.34
**Extraversion**	0.10
**Agreeableness**	0.42
**Neuroticism**	0.31
