# Supplementary material for: Childhood adversity and educational attainment: Evidence from Zambia on the role of personality
Source: Front Psychol. 2023 Jan 27;14:995343. doi: 10.3389/fpsyg.2023.995343 (PMC9912843; doi:10.3389/fpsyg.2023.995343)
Supplement: Supplementary file 5 [file Table_5.pdf]

Table S5 Correlations between personality traits

	**Openness**	**Conscientiousness**	**Extraversion**	**Agreeableness**	**Neuroticism**
**Openness**	1.00				
**Conscientiousness**	0.25	1.00			
**Extraversion**	-0.02	0.02	1.00		
**Agreeableness**	0.34	0.39	0.04	1.00	
**Neuroticism**	0.21	-0.01	0.09	0.07	1.00
